# Type-2 Diabetes Mellitus and the Gut Microbiota: Systematic Review

**DOI:** 10.7759/cureus.49740

**Published:** 2023-11-30

**Authors:** Ethan Slouha, Atbeen Rezazadah, Kiana Farahbod, Andrew Gerts, Lucy A Clunes, Theofanis F Kollias

**Affiliations:** 1 Pharmacology, St. George's University School of Medicine, St. George's, GRD; 2 Pharmacology, St. George's University School of Medicine, St. George, GRD; 3 Pharmacology, St. George's University, St. George's, GRD; 4 Microbiology, Immunology and Pharmacology, St. George's University School of Medicine, St. George's, GRD

**Keywords:** metabolism, bacteria, influences of gut microbiota, type 2 diabetes mellitus, gut microbiota

## Abstract

The gut microbiota is a community situated in the gastrointestinal tract that consists of bacteria thriving and contributing to the functions of our body. It is heavily influenced by what individuals eat, as the quality, amount, and frequency of food consumed can favor and inhibit specific bacteria. Type-2 diabetes mellitus (T2DM) is a common but detrimental condition that arises from excessive hyperglycemia, leading to either insulin resistance or damage to the B-cells that produce insulin in the pancreas. A poor diet high in sugar and fats leads to hyperglycemia, and as this persists, it can lead to the development of T2DM. Both insulin resistance and damage to B-cells are greatly affected by the diet an individual consumes, but is there a more involved relationship between the gut microbiota and T2DM? This paper aimed to evaluate the changes in the gut microbiota in patients with T2DM and the impacts of the changes in gut microbiota. *Bacteroides, Proteobacteria, Firmicutes,* and *Actinobacteria* prevailed in patients with T2DM and healthy control, but their abundance varied greatly. There was also a significant decrease in bacteria like *Lactobacilli *spp.and *F. prausnitizii* associated with insulin resistance. High levels of BMI in patients with T2DM have also been associated with increased levels of *A. muciniphilia, *which has been associated with decreased fat metabolism and increased BMI. Metabolites such as butyrates and melatonin have also been identified as influencing the development and progression of T2DM. Testosterone levels have also been greatly influenced by the gut microbiota changes in T2DM, such that males with lower testosterone have a greater abundance of bacteria like *Gemella, Lachnospiraceae,* and *Massiia.* Identifying these changes and how they impact the body may lead to a treatment addressing insulin dysfunction and the changes that the altered gut microbiota leads to. Future research should address how treatment methods such as healthy diets, exercise, and anti-diabetics affect the gut microbiota and see if they influence sustained changes and reduced hyperglycemia.

## Introduction and background

The gut microbiota

The gut microbiota consists of numerous microorganisms, including bacteria, fungi, archaea, viruses, and protozoans [1]. This microbiota is associated with various human diseases, such as luminal conditions and metabolic disorders. These non-pathological microorganisms play an essential role in nutrient and drug metabolism, preventing pathogenic colonization, and maintaining intestinal barrier function, all while coexisting symbiotically with the host’s immune system [1]. The predominant phyla in the gut microbiota have been reported to be Firmicutes and Bacteroidetes, with Proteobacteria, Firmicutes, Actinobacteria, and Bacteroidetes being the most abundant microbes in the gut. However, their relative abundance varies significantly across individuals. Additionally, the gut microbiota differs spatially along the gastrointestinal tract [1,2].

The gut microbiota contributes various benefits to the host, including maintaining mucosal barrier integrity, providing nutrients, and protecting against pathogens. These microbes produce short-chain fatty acids (SCFAs), essential for regulating cellular processes, energy metabolism, immune response, and epithelial health. SCFAs also regulate the host’s metabolic state, altering appetite. The gut microbiota helps maintain epithelial homeostasis, promoting cell renewal, wound healing, and mucus properties, ultimately influencing the competitive advantage of certain commensal species in the gut. The gut microbiota affects the development and function of the host’s immune system, inflammatory gut disorders, IgA responses, and immune-stimulatory capacities. It plays a crucial role in protecting against pathogen colonization by competing for attachment sites and inducing the host to produce various antimicrobial compounds, including antimicrobial peptides (AMPs) and secretory IgAs. Thus, it helps protect against pathogens and maintains a beneficial relationship with commensal bacteria [2].

Influences of gut microbiota

Diet significantly influences the composition of the gut microbiome, as the bacteria that constitute the microbiome utilize dietary nutrients from the host for their energy and nutritional needs, starting in infancy [2,3]. Gestational age at birth has significant implications for gut microbiota, with preterm infants showing decreased levels of diversity and more colonization by potentially pathogenic bacteria in their gut microbiomes than term infants [4]. Complex carbohydrates are an essential food source for the commensal bacteria in the gut microbiome. The Western diet, containing many heavily processed foods often lacking in fiber and complex carbohydrates, negatively impacts the amount and quality of food available for commensal microbes [3].

Human microbiomes can be categorized into two large groups called enterotypes: Bacteroides-dominant or Prevotella-dominant. These enterotype classifications can be inferred based on diet [3]. Enterotype classifications of human gut microbiota are rooted in long-established dietary patterns and typically remain stable throughout an individual’s life. Bacteroides-dominant enterotypes are significantly associated with diets high in animal fats and protein, while Prevotella-dominant enterotypes are substantially associated with diets high in carbohydrates [3]. The composition of gut microbiomes differs between individuals due to factors such as body mass index, exercise frequency, lifestyle factors, cultural habits, and dietary intake. Human gut microbiota vary in different parts of the gastrointestinal tract, both functionally and taxonomically. Variations within a single individual can be attributed to transitions in infancy, age, diet, antibiotic use, and many other external factors. Dysbiosis within the gut microbiome is strongly associated with metabolic, neurological, and intestinal disorders [4].

Type-2 diabetes mellitus

Type-2 diabetes mellitus (T2DM) is one of the most common metabolic diseases worldwide, affecting over 35 million people in the United States alone. T2DM can occur due to inadequate insulin production from pancreatic B-cells or tissues in the body becoming insulin resistant. B-cell dysfunction is primarily caused by B-cell death, which can occur in different ways; however, it is mainly due to the accumulation of reactive oxygen species (ROS) and increased inflammation. Over time, B-cells become predisposed to increased inflammation and toxins, such as ROS and amyloids. This ultimately leads to structural deformities, resulting in the loss of islet integrity. Excess free fatty acids and hyperglycemic conditions can lead to B-cell dysfunction by inducing ER stress and activating pro-apoptotic pathways, impairing ER homeostasis. Prolonged hyperglycemia can increase insulin synthesis and islet amyloid peptides within B-cells, accumulating improper and misfolded proteins. These improper proteins cause the body to create more ROS, further damaging the ER [5].

Another mechanism is prolonged hyperglycemia from diet, which can also result in the desensitization of GLUT4 receptors, preventing them from recognizing insulin and rendering the tissues and organs resistant to the effects of insulin. Insulin resistance can occur due to mutations in the tyrosine kinase or glycolysis pathways. The tyrosine kinase pathway is essential for activating insulin production. The alpha subunit on the tyrosine kinase receptor plays a vital role in insulin-mediating signaling [6]. Mutations in tyrosine kinase receptors have a downstream effect, causing increased insulin resistance and decreased glucose absorption into the muscles [6]. The most common symptoms of T2DM are increased thirst, polyphagia, and polyuria, due to the blood sugar imbalance and loss of homeostasis between the brain and the kidneys [7]. The high sugar levels in the bloodstream force the kidneys to work harder to excrete the excess sugar. Thus, more urine is produced as water is pulled from tissues into the urine, increasing urination and the need to hydrate [7, 8].

Aim

It is well known that most cases of T2DM are due to the overabundance of fats and sugars in the diet, which is also seen in the Western diet. At the same time, the Western diet consisting of high fat and high sugar is shown to drastically alter the gut microbiota of individuals compared to those with a more balanced diet. It has also been shown that this dysregulation of diet can lead to the overgrowth or reduction of critical bacteria such as Firmicutes or Bacteroides and bacteria that create essential metabolites such as SCFAs, which then, in turn, affect what our body can produce or our how body functions. This paper aims to analyze how the gut microbiota affects the development of, and to some extent, as a patient develops, T2DM.

## Review

Methods

A methodical and calculated literature review was accomplished using three electronic databases from January 1, 2013, to December 31, 2023: PubMed, ScienceDirect, and ProQuest. The keywords for the search were ‘type 2 diabetes and gut microbiota’, ‘t2dm and gut microbiota’, ‘changes in gut microbiota and t2dm’, and ‘changes in gut microbiota and type 2 diabetes’. The investigation concentrated on peer-reviewed experimental and observational publications. Studies that were duplicates, not written in English, and published before 2013 were excluded from the review. After the procurement, publications were evaluated on their title, study, abstract, and full-text availability. The preliminary analysis of the used databases resulted in 66,064 publications. The data presented in the abstract of the publication were cross-referenced with the keywords leading to a specific amount of selected publications to address the aim of this publication. A total of 29 publications were collected according to the following criteria.

Inclusion Criteria

The publications were chosen based on the following inclusion criteria: publications between 2013 and 2023, performed on humans, focused on gut microbiota for T2DM without major influences of anti-diabetes, peer-reviewed experimental or observational studies, and full-text.

Exclusion Criteria

**Figure 1:**
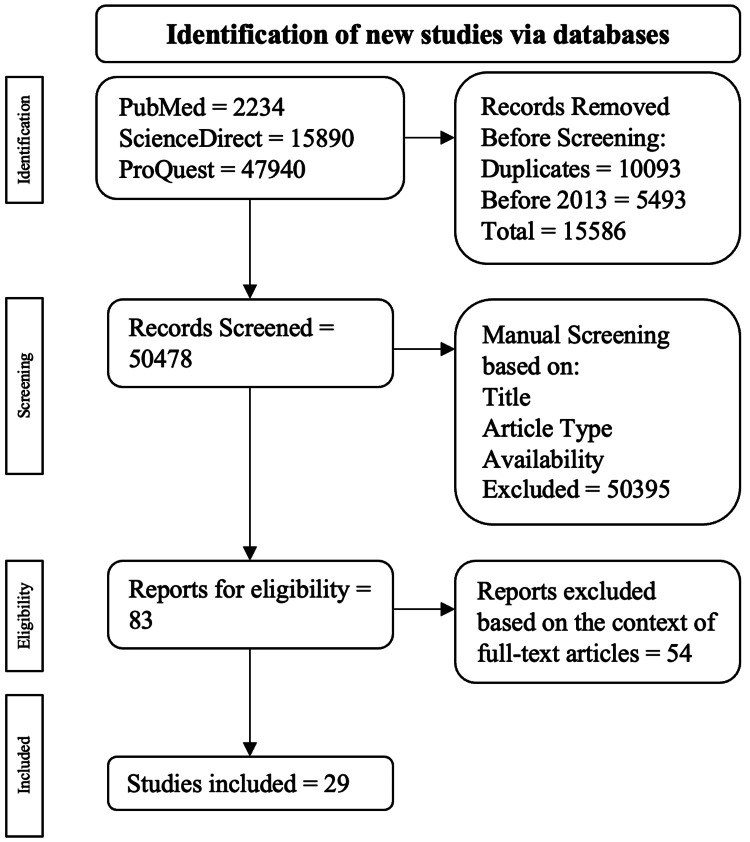
Visual representation of the inclusion and exclusion criteria progression. The pathway used was adapted from the PRISMA review [9].

The publications were excluded based on the following exclusion criteria: duplicated articles, articles not written in English, and not full-text publications. The process of selecting publications through inclusion and exclusion criteria is drawn out in Figure 1.

Bias

All publications were assessed for bias via the GRADE (grading of recommendation, development, and evaluation) scale, resulting in a moderate bias rating. The bias tool GRADE was selected as it weights errors in publications like the publication, indirection, and imprecision.

Results

A total of 47,940 publications were discovered: 2234 were from PubMed, 15,890 were from ScienceDirect, and 47,940 were from ProQuest. Among the exclusions, 10,093 were duplicate publications, and 5493 were published before 2013. This resulted in 15,586 publications being excluded during the automatic screening procedure, leading to 50,478 publications for manual screening. Publications were then manually evaluated based on their full-text accessibility, title, and type of study, resulting in 54 publications being verified for eligibility through full-text analysis. Ultimately, 29 publications were selected.

*Bacteriodetes, Firmicutes, Proteobacteria*, and *Actinobacteria* were widely abundant in patients with T2DM and healthy controls; however, some studies have found that there’s a significant increase in the *Firmicutes:Bacteroidetes* ratio due to a significant increase in *Firmicutes*. In patients with T2DM, there’s a substantial decrease in alpha- and beta-diversity. There’s also a decrease in *F. prausnitizii*, correlated with anti-inflammatory markers, and a reduction in *Clostridium*, positively associated with decreasing insulin resistance. BMI is also reduced in T2DM, associated with the increased *Fsirmicutes:Bacterioidetes* ratio. Also, the elevated levels of *A. muciniphilia* observed in T2DM are associated with increased BMI, possibly due to decreasing levels of fat metabolism. T2DM also has a reduction in butyrate-producing bacteria, which produce SCFAs and is also associated with insulin resistance and reduced insulin production. Studies have also postulated that excessive DNA methylations in the gut can predispose an individual to T2DM, leading to higher fasting blood glucose and HbA1c levels. As well as what was discussed, *Firmicutes* have been significantly correlated with increased fat storage in patients with T2DM. Lastly, the gut microbiota in males with T2DM can lead to significantly reduced testosterone concentration levels compared to healthy controls. The results acquired were based on analysis of selected articles, and these articles are summarized in Table 1.

Discussion

Similarities in Gut Microbiota

Both patients with T2DM and healthy controls had similarities in their gut microbiota composition at the phylum and class level as well as their Choa1 richness, PD_whole tree, and to extent alpha-diversity in one study [10-13]. Regarding phylum levels, there was a prominent abundance of *Bacteroidetes, Firmicutes, Proteobacteria*, and *Actinobacteria* in healthy controls and patients with T2DM [12,14-17]. At the class level, there was also an abundance of *Bacteroidia, Actinobacteria, Clostridia, Bacilli, Gammaproteobacteria*, and *Betaproteobacteria* [12]. While the predominance of these microorganisms compared to others is similar in both groups, the specific amount of each varies between each group. Xiang et al. determine that mechanisms like improved glucose metabolism, enhanced insulin sensitivity, and altered intestinal permeability contribute to the link between T2DM and the gut microbiota [13].

Differences in Gut Microbiota

It is observed that increased levels of glucose in the blood are associated with decreased levels of overall gut microbiota diversity, and patients with T2DM were observed to have reduced richness of their gut microbiome composition [11,17-19]. There is a question as to whether the microbiome could be a valuable tool in the risk evaluation of the development of T2DM and several bacterial taxa correlated with risks, such as the prevalence of Granulicatella and Prevotella [18]. Neri-Rosario et al. observed that predictors of patients with preT2DM were higher relative abundances of *Escherichia, Shigella, Collinsella, Senegalimassilia, *and *Allisonella*, and a decrease in abundance of *Enterococcus, Intestinibacter, *and *Anaerostipes* [20].

In healthy controls, it was observed that at least 87 operational taxonomic units oscillate daily. In contrast, in patients with T2DM, it was found to be arrhythmic without *Bacteroidetes* and *Firmicutes* [14]. In patients without T2DM, *Actinobacteria* was prevalent at the phylum levels and the class level, *Clostridiaceae, Porphyromonadaceae*, and *Ruminococcaceae* families [18], whereas in patients with T2DM, *Bacilli* and *Negativicutes* classes were prevalent, and the *Lactobacillales* and *Selenomonadales *orders, as well as *Dorea* and *Roseburia* [18,21]. In patients with T2DM, there’s also a significant decrease in alpha- and beta-diversity observed in most studies [16,22-24].

In patients with T2DM, there was discord in the abundance and ratios of *Bacteroidetes* and *Firmicutes*, some studies accounting for their differences as dysbiosis within the gut microbiota. A few studies have found a significant decrease in *Bacteroidetes* and a correlated increase in the *Firmicutes:Bacteroidetes *ratio [10,12,17,25]. Compared to three studies that found an increase in *Bacteroidetes* and a correlated decrease in the *Firmicutes:Bacteroidetes* ratio [11,21]. The majority of studies, however, did observe a significant increase in the abundance of *Firmicutes* and a growth in the *Firmicutes:Bacteroidetes* ratio [10,17,21,26,27]. One study found that Firmicutes abundance was reduced [25]. Studies found that this signified the gut microbiota difference between patients with and without T2DM as a common trend; however, there are more disturbances to the gut microbiota.

In patients was T2DM there was a significant reduction in the abundance of *Lactobacilli spp, Clostridium cluster IV, Faecalibacterium prausnitizii (F. prausnitizii), Bifidobacterium, Verrucomivrobia, Proteobacteria, Elusimicrobia, Prevotella stercorea, Veillonella dispar *and *parvula, Roseburia faecis, Enterobacterieaceae, Erysipelotrichacaea, Peptostreptocacceae*, and *Tenericutes *[10,12,15,22,28-30]. The decrease in *F. prausnitizii* may be vital as it is considered an anti-inflammatory marker, and the decreased levels of *Clostridium* and *Anaerostipes* have been positively associated with reducing insulin resistance [23,29]. There was also a significant reduction in butyrate-producing microorganisms in patients with T2DM, such as *F. prausnitizii, Ruminococcus, Clostridium leptum, Butyricicoccus, Alistipes spp., Pseudoflavonifractor spp.,* and *Oscillibacter spp.* [19].

While some studies have noticed a reduction in *Bifidobacterium, Prevotella,* and *Lactobacillales* in patients with T2DM, more studies have noted a significant increase in abundance [11,12,15,16,24,31]. There’s also a substantial increase in the quantity of *Proteobacteria, Fusobacteria, Dorea, Succinibrionaceae, Lanchnospiraceae, Blautia producta, Haemophilus parainfluenczar, Ruminococcus gnavus, Neisseria, Akkermansia, Acidaminococcus, Megamonas, Dialister, Actinobacteria, Streptococci, Spirochaetes, Acarbose, Salmonella spp., Campylobacter spp., Yersina enterocolitica, Candida albicans*, and *Shigella spp*. patients with T2DM [11,12,16,17,22,24,25,28,31-33]. There’s also an increase in abundance in some butyrate producers, specifically *Clostridium bolteae* [19].

Fecal Differences

Fecal microbiota varied greatly in patients with T2DM and was associated with gut microbiota, but research did not agree on the direction of variability. One study found that fecal levels of cholic acid, glycoursodeoxycholic acid, gylcocholic acid, and *Proteobacteria* families, specifically beta and gamma, were significantly increased [34]. Other studies found significantly reduced levels of *B. fragilis*, cholic acid, glycocholic acid, and glycoursodeoxycholic acid [17,30]. Zhao et al. observed that cholic acid might play a role in developing complications in patients with T2DM, as those with disease-related complications had significantly reduced levels [17]. It was observed, however, that the time of defecation did play a significant role in explicating individual variation in the composition of gut microbiota, such as *Bacteroidetes* was more abundant at night while *Firmicutes* was more abundant during the daytime [14].

BMI Association With Gut Microbiota

Studies have also focused on the contribution that the BMI of patients with T2DM has on the gut microbiota. One study observed that patients with higher BMIs had increased *Firmicutes:Bacterioidetes* ratio [27]. Another study found a nonsignificant positive correlation between BMI and the abundance of the *Fusobacterium* and *Lactobacillus* groups [15]. There was also no significant correlation between *B. longum, B. fragilis, F. prausnitzii,* and *Prevotella* and BMI [15,30]. However, Carrizales-Sanchez et al. observed a direct positive correlation between the levels of *Prevotella* and BMI [31]. Fassatoui et al. observed elevated levels of *A. muciniphilia* in T2DM, which was functionally associated with decreased fat metabolism and increased BMI levels [29].

Metabolites in Gut Microbiota

Several metabolites and hormones have been observed to play a unique role in the development and continuance of T2DM. There was a lower risk of T2DM in patients with higher levels of serum melatonin; this was more prominent in men than women [24]. This further correlated to a significant association between higher serum melatonin levels and lower fasting plasma glucose levels [24]. This may stem from the tryptophan metabolism pathway, as there is a significant difference between patients with and without T2DM [24]. In patients with T2DM, there was a correlation between *Coprococcus* and *Bifidobacterium* and melatonin-related metabolites and inflammation factors [24]. Using this analysis, Huang et al. identified potential predictors of T2DM, such as 2-oxomelatonin, 5-hydroxyindoleacetylgylcine, and tryptophanol. They were negatively correlated with *Coprococcus*, while 5-hydroindoleacetylgylcine and tryptophanol were positively correlated with *Bifidobacterium* [24].

Another influence of T2DM on gut microbiota is its impact and alteration of butyrates and short-chain fatty acids (SCFA). There were also variations in the observed levels of SCFAs where one study found that there were significantly increased levels of propionate, acetate, and isovaleric acid, and another found decreased levels of acetate, hexanoic acid, propionate, valeric acid, and butyrate [17, 34]. In pre-T2DM and T2DM patients, nearly half of the metagenomic species that experienced significant changes in abundance, most being reduced, were potential producers of butyrate such as *Akkermansia, Prevotella, Roseburia, Faecalibacterium spp., Clostridium spp., Alistipes spp., Pseudoflavonifractor spp*., and *Oscillibacter spp.* [19,21,29]. Patients with T2DM displayed decreased butyrate-producing bacteria, which has been associated with insulin resistance [23]. *Akkermansia* and *Prevotella* are associated with an increased production of butyrate, which is a beta cell activator, and this is decreased in patients with T2DM, also decreasing insulin production [29]. *Dorea*, which is associated with obesity, was increased in patients with T2DM, and this correlated with a reduction in butyrate-producing bacteria [21].

Specific metabolites are significant predictors in the development of T2DM, such as 1-linoleoyl-glycerophosphocholine, creatine, and indolepropionic acid (IPA) [35,36]. Patients who progressed to impaired glucose tolerance had lower levels of IPA, directly associated with insulin secretion [35]. There was also a positive correlation between serum IPA and dietary fiber intake, whereas a negative correlation was found between serum IPA and serum high-sensitivity C-reactive protein [35]. Serum imidazole propionate (ImP) was also significantly higher in patients with pre- and T2DM [37]. Serum ImP was negatively correlated with Diversity Score, Mediterranean diet scores, and Healthy Eating Index [37]. Low microbial diversity and *Bacteroides 2 enterotype* linked to inflammatory bowel disease and obesity are also associated with ImP levels [37]. It is also observed that ImP production may be partly due to an unhealthy diet, as it is increased with an unhealthy diet [37]. The reduction of ImP due to an unhealthy diet seen in most patients with T2DM may contribute to the dysbiosis of the gut microbiota in patients with T2DM [37].

Significance of Gut Microbiota on T2DM Mellitus

Excessive DNA methylation can occur in patients with varying gut microbiota levels, predisposing obese individuals to T2DM [32]. *Eubacertialis, Clostridia,* and *Firmicutes* increase DNA methylation, which confers with their positive association with the development of T2DM [32]. This higher methylation also led to higher fasting blood glucose and HbA1c levels [32]. Different bacteria were also associated and influenced with the low-density lipoprotein (LDL) and high-density lipoproteins (HDL) levels in patients with T2DM and fat storage. Elevated levels of *Lactobacillus* were positively associated with higher levels of LDL and *Oscilopsia* and had negative correlations with LDL levels in patients with T2DM [31]. There was also a significant correlation between *Firmicutes* and increased fat storage in patients with T2DM [26]. Regarding glucose levels, decreased levels of *Proteobacteria* correlate with increased proinflammatory cytokines and impaired pancreatic beta-cell function, leading to high blood glucose [28]. *Faecalibacterium* levels positively correlated with increased glucose levels in patients with T2DM [31]. This could be due to the observation of drastically decreased cell signaling leading to reduced carbohydrate and amino acid metabolism levels in patients with T2DM [28].

A complication that can further cause gut dysbiosis and a “leaky gut” in patients with T2DM is the alterations of zonula occludens-1 (ZO-1) due to inappropriate levels of gut microbiota. *Bacteroides* and *Prevotella 9* were found to have negative correlations with ZO-1 and with levels of acetate, propionate, and serum total SCFAs [34]. Due to the changes in the gut microbiota, there’s a significantly increased level of ZO-1 in patients with T2DM, indicating a “leaky gut”. This further shows that ZO-1 positively correlates with BMI, fasting blood glucose, HbA1c, and systolic and diastolic blood pressure, which relates to symptoms associated with T2DM [34]. A couple of random correlations were also observed in Guo et al.’s study. Increased levels of oral *Streptococci* were seen in patients with T2DM, which has been shown to increase the chance of oral disease. Increased levels of *Actinomyces* have been shown to lead to the accumulation of intracellular polysaccharides and an increase in the chances of developing gingival disease. Also, increased periodontal disease and gingivitis levels were closely associated with increased *Spirochaetes* and *Proteobacteria* [33].

Studies have also found an effect on testosterone levels regarding varying gut microbiota. Male patients with low testosterone had a greater abundance of *Massilia, Gemlla, Lachnospiraceae, Actinoplanes, Allorhizobium, Neorhizobium, Parahizobiu, Solobacterium, Lachnoclostridium, Parvimonas, Bergeyella, *and *Blautia* [38]. At the same time, males with normal testosterone had a greater abundance of *Candidatus, Saccarimonas, Paludicola,* and *Allisonella* at the genus level [38]. Intestinal bacteria that showed the greatest association with low testosterone levels were *Butyricicoccus, Blautia, CAG-56 Lachnoclostridium, Actinomyces, Fusicatenibacter, Bergeyella, Streptococcus,* and *Solobacterium* [38]. While there are significant differences in the gut microbiota of patients with and without T2DM, this difference may also play a role in male patients who suffer from low testosterone and T2DM.

A primary limitation of this study is that most studies did not compare or evaluate the effects of oral antidiabetics on the gut microbiota used to treat T2DM. The therapy can significantly influence the balance of gut microbiota in these patients as beneficial bacteria may be increased. While there is a difference in the gut microbiota between healthy individuals and those with T2DM, these results may not be as accurate as they should be. There is also a lack of studies on how these significant differences affect the body. Still, there is where future research should focus so that treatments can stop this effect and treat the imbalance.

**Table 1:** Summary of articles used for analysis in this review T2DM: type 2 diabetes mellitus.

S. No.	Author	Country	Design and Study Population	Findings	Conclusion
1	Ahmad et al., 2019 [10]	Pakistan	Cohort study (n = 60)	Statistical testing presented no difference for the PD_whole tree and Choal richness. The Firmicutes phyla were increased from 55.7% to 36.9% in those with T2DM, while Verrucomivrobia, Bacteriodietes, Proteobacteria, and Elusimicrobia were reduced or absent in obese patients.	This study showed a correlation between the individual lifestyle of patients with T2DM and how their lifestyle can alter their gut microbiota.
2	Al-Muhanna et al., 2022 [11]	Saudi Arabia	Cohort study (n = 440)	Those with T2DM had a statistically different biome arrangement compared to those without T2DM. Elevated Bacteroidetes:Firmicutes ratio levels were present in those with T2DM. Akkermansia, Acidaminococcus, Megamonas, Dialister, Lactobacillus, and Paraprevotella were enriched in those with T2DM.	This study has helped assess this region's current health epidemic and shown the microbiota's importance. Additionally, it showed that some bacteria are more prevalent in those with T2DM.
3	Kulkarni et al., 2021 [12]	India	Cohort study (n = 10)	There were as higher amounts of inflammatory bacteria such as Lactobacillus ruminis, Bacteroides cacae, and Butyricimonas, and a lower amount of anti-inflammatory bacteria suck as Butyrivibrio and Faecalibacterium prausnitzii which likely play a role in the development of T2DM.	Diagnostic markers or possible therapeutic targets to treat T2DM may focus on the increased inflammatory and decreased anti-inflammatory bacteria present.
4	Xiang et al., 2022 [13]	China	Experimental study (n = 18,473)	The study did not find significant evidence of a causal relationship between various gut microbiome families and T2DM risk, although borderline positive correlations were observed for Streptococcaceae and Acidaminococcaceae.	The study found borderline positive correlations between Streptococcaceae and Acidaminococcaceae in the gut microbiome and T2DM risk.
5	Reitmeier et al., 2020 [14]	Germany	Cohort study (n = 4,132)	The composition of the human gut microbiome demonstrates a diurnal rhythm, and in obese people and patients with T2DM, this circadian rhythm is deranged. Arrhythmia in the signatures of gut microbiota allows for the classification of risk in predicting the development of T2DM.	Individual gut microbiota compositions can be significant risk factors for developing metabolic disorders such as T2DM. A potential functional association was found between circadian rhythms and gut microbiota and their intertwined roles in the development of metabolic disease.
6	Sedighi et al., 2017 [15]	Iran	Case-control study (n = 36)	Bifidobacterium was significantly more abundant in healthy individuals compared to patients with T2DM. Lactobacillus was significantly more abundant in patients with T2DM compared to healthy individuals. There was no significant difference between the groups in the levels of Prevotella and Fusobacterium.	The dysbiosis of the gut microbiota in patients with T2DM may provide a way to facilitate the treatment of the disease.
7	Takagi et al., 2020 [16]	Japan	Case-control study (n = 239)	Significant structural differences in the fecal microbes between individuals with the alpha diversity between the groups varied significantly. More specifically, levels of Alistipes were lesser in patients with only hyperlipidemia, and levels of Bifidobacterium were significantly higher in patients with hyperlipidemia comorbid with T2DM.	Although, structurally, the gut microbes between healthy controls and patients with two comorbid conditions (T2DM, hypertension, hyperlipidemia) did not differ significantly, the alpha-diversity and metabolic enzymes differed significantly.
8	Zhao et al., 2019 [17]	China	Case-control study (n = 65)	In patients with T2DM, gut microbiome composition and fecal metabolite profiles were significantly different from healthy controls. Disordered levels of short-chain fatty acids, bile acids, and lipids were noted in patients with T2DM, as well as a higher level of Proteobacteria and a greater ratio of Firmicutes:Bacteroidetes.	A relationship exists between gut microbiome composition and fecal metabolite profiles in patients with T2DM who are not currently treated with metformin.
9	Vals-Delgado et al., 2022 [18]	Spain	Cohort study (n = 462)	Using a predictive model based on the microbiome, an association was determined between a profile of gut microbiota and the development of T2DM. Clinical parameters such as HbA1c, HDL, the Finnish T2DM Risk Score, triglyceride levels, and the T2DM risk score of the American T2DM Association were added to the microbiome data, thus enhancing the predictive capabilities of the microbiome-based predictive model.	There may be a specific profile of gut microbiota associated with the development of T2DM. Additionally, the prediction of T2DM development can be improved by using a predictive model that combines microbiome data and clinical data, which may enable more successful disease prevention.
10	Wu et al., 2020 [19]	Denmark, Sweden	Cohort study (n = 1495)	No significant changes were observed in the composition of gut microbiota in participants with impaired fasting glucose. In contrast, changes were observed in those with impaired glucose tolerance and glucose intolerance combined with T2DM. In participants with preT2DM and T2DM, a decrease in butyrate production and several butyrate producers were observed.	In subjects of varying glycemic status, naïve to T2DM treatment, it was found that alterations in gut microbiota are associated quite powerfully with levels of insulin resistance.
11	Neri-Rosario et al., 2023 [20]	Mexico	Cohort study (n = 410)	Dysbiosis in gut microbiota plays a causal role in the development and progression of T2DM. Specific taxa were elucidated and found to predict subjects who had T2DM and preT2DM and delineate them from healthy, normoglycemic controls.	There appears to be a distinctive composition and bacterial signature of gut microbiota in patients with T2DM, and this information can be used in the prevention and treatment of the disease in new ways.
12	Esquivel-Hernandez et al., 2023 [21]	Mexico	Network analysis	This paper underscores the alterations in individuals' gut microbiota compositions that play a role in the development of T2DM. However, it is essential to note that this study does emphasize genera such as Prevotella and Blautia as significant players in the progression of T2DM.	The relationship found is that specific bacterial genera in the gut microbiota could potentially serve as biomarkers to differentiate the stages of progression in T2DM within the Mexican patient cohort.
13	Balvers et al., 2021 [22]	Netherlands	Cohort study (n = 452)	Patients with T2DM showed drastic reconstruction of their gut microbiome, with increased levels of Escherichia/Shigella and decreased levels of Peptostreptocacceaea.	A positive relationship was also discovered between ethnicity and levels of specific gut flora.
14	Doumatey et al., 2020 [23]	Africa	Cohort study (n = 291)	The beta diversity in the gut microbiome of patients with T2DM seemed to be significantly altered compared to the control group. Patients with T2DM displayed decreased butyrate-producing bacteria, which has been associated with insulin resistance.	Specific biomarkers were significantly decreased in T2DM, such as butyrate-producing bacteria associated with insulin resistance.
15	Huang et al., 2022 [24]	China	Case-control study (n = 2034); case-control study (n = 120)	Increased melatonin levels were associated significantly with lower levels of FBG and lower risk of T2DM. In patients with T2DM, there were lower levels of melatonin, a- and B- diversity of microbiota, lower abundance of Coprococcus, and greater abundance of Bifidobacterium. This was correlated with altered levels of inflammatory markers.	High melatonin levels in serum were significantly associated with a decreased risk of T2DM. Bifidobacterium and Coprococcus are involved in the melatonin signaling with tryptophan.
16	Polidori et al., 2022 [25]	Italy	Cohort study (n = 334)	In patients with T2DM, the ratio of Bacteroidetes to Firmicutes was decreased due to the eating habits of the study participants, and an increased gut permeability was observed in female patients especially.	In the Italian population, T2DM was statistically significantly associated with specific dysbiosis within the gut microbiota.
17	Cai et al., 2015 [26]	China	Cohort study (n = 1121)	European patients with T2DM showed increased levels of fat storage markers. Both European and Chinese patients with T2DM had elevated levels of Glycan biosynthesis. EggNOG levels were increased in Europeans.	A positive relationship was shown between EggNOG levels in patients with T2DM. Additionally, a positive relationship was demonstrated in KEGG marker levels in patients with T2DM.
18	Hung et al., 2021 [27]	Taiwan	Cohort study (n = 179)	Patients with T2DM also had higher levels of Firmicutes to Bacterioidetes. They noticed that the Firmicutes:Bacterioidetes ratio increased as BMI levels increased.	This study helped show that specific gut microbiota is increased in patients with T2DM and BMI.
19	Du et al., 2022 [28]	China	Case-control study (n=73)	T2DM displayed low levels of specific gut microbiota. These decreased levels have been associated with increased levels of proinflammatory cytokines and impaired pancreatic beta-cell function. Decreased levels in Lactobacillaceae and Erysipelotrichacaea are associated with lower levels of carbohydrate metabolism and a decrease in amino acid metabolism.	Decreased levels of Tenericutes have been shown to interfere with beta-cell secretion and increased levels of inflammation.
20	Fassatoui et al. 2019 [29]	Africa	Case-control study (n=11)	Elevated levels of A. muciniphilia were seen in patients with T2DM, associated with decreased fat metabolism and increased BMI levels. Patients with T2DM also had reduced levels of Akkermansia and Prevotella, resulting in decreased production of butyrate and insulin.	T2DM displayed decreased levels of Akkermansia and Prevotella, which have been shown to reduce beta cell production, resulting in insulin resistance.
21	Navab-Moghadam et al., 2017 [30]	Iran	Case-control study (n = 36)	While Faecalibacterium prausnitzii was significantly decreased in patients with T2DM, Bacteroides fragilis showed a decreased trend in presence, but it was not significant. B. longum's abundance did not significantly differ between patients with and without T2DM.	The decrease in Faecalibacterium prausnitzii and Bacteroides fragilis may be potential therapeutic targets in T2DM.
22	Carrizales-Sanchez et al., 2023 [31]	Mexico	Cohort study (n = 66)	Significantly higher levels of Lanchnospiraceae were found in patients with T2DM. Prevotella and Dorea were elevated, while normal levels were observed in the control group. The control group had significantly lower levels of Oscillopsia, which showed a significant negative correlation with LDL levels.	Specific biomarkers were found to be positively associated with increased chances of developing hypercholesterolemia as well as developing obesity in those patients with T2DM.
23	Guo et al., 2022 [32]	China	Cohort study (n = 29)	This study showed that certain bacterial groups, such as Eubacertiales, Clostridia, and Formicutes, have been positively associated with T2DM. Eubacertiales, Closteridia, and Formicutes had higher methylation levels than the beneficial gut microbiota. Additionally, the individuals who had Eubacertiales, Closteridia, and Formicutes present in their gut microbiota also had higher levels of resting blood glucose and HbA1c levels.	This study helped show that DNA methylation may be related to T2DM. It showed that there were higher rates of developing T2DM in bacteria that underwent DNA methylation than in bacteria that did undergo DNA methylation.
24	Guo et al., 2023 [33]	China	Case-control study (n = 429)	Increased levels of oral Streptococci were seen in T2DM, which has increased the chance of oral diseases, such as periodontitis. Increased levels of Actinomyces have been shown to lead to the accumulation of intracellular polysaccharides and increase the chances of developing gingival diseases.	T2DM showed an increase in a wide variety of oral bacteria, specifically in Streptococci and Actinomyces. It was discovered that Actinomyces can increase fat accumulation, resulting in higher BMI levels.
25	Zhao et al., 2020 [34]	China	Case-control study (n = 100)	Patients with T2DM had significantly higher levels of short-chain fatty acids, acetate, propionate, and specific bile acids compared to the healthy controls. It was also found that the patients with T2DM had increased levels of Zonula Occludens-1, or ZO-1, associated with a "leaky gut" in this patient population.	In patients with T2DM, dysbiosis of the gut microbiome may lead to a leaky gut, where short-chain fatty acids and bile acids are absorbed intestinally in excessive amounts, leading to high circulating concentrations of short-chain fatty acids and bile acids in this patient population.
26	Tuomainen et al., 2018 [35]	Finland	Randomized control study (n = 403)	Patients in the lifestyle intervention group had higher serum indolepropionic acid levels at the 1-year point of the study, which was associated inversely with T2DM incidence and directly with insulin secretion. A positive correlation was found between dietary fiber intake and serum indolepropionic acid, while a negative correlation was found between high-sensitivity C-reactive protein concentrations and IPA at sampling and study follow-up.	Serum indolepropionic acid, a metabolite of microbiota in the gut, was effective in lowering risk in patients at high risk of developing T2DM. This suggested effect of IPA may be mediated by the interactions and associations between inflammation and dietary fiber intake or, more directly, by IPA's effect on pancreatic β-cell function.
27	Vangipurapu et al., 2020 [36]	Finland	Cross-sectional study (n = 5,181)	Levels of specific metabolites, including creatine, urate, and xanthine, among others, were associated significantly with a greater risk of the development of T2DM.	The microbial metabolites found to be significantly associated with an increased risk of T2DM have a great and presently underemployed utility in being used as biomarkers for elucidating the risk of developing T2DM.
28	Molinaro et al., 2021 [37]	Sweden	Population study (n = 1990)	ImP levels were increased in patients with T2DM, which was associated with a low abundance of microbial diversity and a specific gut microbiome enterotype. The study also found that an unhealthy diet, but not histidine intake, was associated with increased ImP levels, suggesting that an unhealthy diet can change the microbial environment in the gut and increase the production of ImP.	An unhealthy diet may contribute to an altered microbial community type with increased potential to metabolize dietary histidine to ImP, which contributes to impaired glucose metabolism by activating MAPK signaling, leading to degradation of insulin receptor substrate and inflammatory signaling.
29	Liu et al., 2022 [38]	UK	Cross-sectional study (n = 146)	The gut microbiome of men with low testosterone levels significantly differed from that of men with normal testosterone levels. Additionally, certain gut bacteria were negatively associated with testosterone levels and positively associated with certain metabolic parameters. The study also found that certain bacteria were negatively correlated with testosterone levels.	Men with low testosterone have significantly different gut microbiota, which correlated with metabolic parameters, meaning that specific bacteria negatives correlate with testosterone levels.

## Conclusions

Patients with T2DM, due to the alteration in the insulin mechanism, lead to excess glucose in the intestines, theoretically altering the environment of the gut microbiota, thereby changing the bacteria involved. While certain microbial groups, such as *Bacteroidetes, Firmicutes, Proteobacteria,* and *Actinobacteria*, prevailed in patients with T2DM and healthy controls, the variation in quantities presented underscored a possible association between T2DM and gut microbiota. Most studies found a significant decrease in *Lactobacilli spp., Clostridium cluster IV, F. prausnitizii*, and *Bifidobacterium*, which have been associated with insulin resistance. Higher levels of BMI were associated with an increase in the *Firmicutes:Bacteroides *ratioand *A. muciniphilia* in T2DM, which was functionally associated with decreased fat metabolism. High levels of melatonin were associated with a reduced risk of developing T2DM, and butyrate was also associated with insulin resistance since butyrate is a B-cell activator. Bacteria producing excessive DNA methylation increased the risk of developing T2DM due to higher levels of FBG and HbA1c. A decrease in Proteobacteria was correlated with impaired beta-cell function, leading to higher blood glucose levels.

T2DM and gut microbiota have an intertwined relationship, with each fueling the imbalance of the other. A few studies observed the trend in gut microbiota as a person develops T2DM and found a significant imbalance in the gut microbiota even before the classification. The relationship is not fully understood and requires more focused research, as understanding this relationship may be the key to treating and curing T2DM. This is because it is known that certain bacteria alter the insulin production and resistance that can lead to T2DM. Once studies identify the true imbalance, treatment can reduce the risk of further complications in T2DM, as mentioned above. Further research focusing on the influences of anti-diabetics, probiotics, and lifestyle modifications should be done to see if they bring about changes to the gut microbiota and how they improve T2DM.
